# Epistemic uncertain computing for intrusion detection with explainability & multi-criteria optimization using AA-NLS and GRUSO-GRU

**DOI:** 10.1038/s41598-026-44214-z

**Published:** 2026-03-18

**Authors:** K. Kiruthika, M. Karpagam, Tanvir H. Sardar, Sk Mahmudul Hassan

**Affiliations:** 1 Department of Mathematics, K.S.Rangasamy College of Technology, Tiruchengode, Namakkal, Tamil Nadu 637 215 India; 2 Department of Computational Intelligence, Faculty of Engineering and Technology, SRM Institute of Science and Technology, SRM Nagar, Kattankulathur, Chennai, Tamil Nadu 603203 India; 3 Department of CSE, School of Engineering, Dayananda Sagar University, Devarakaggalahalli, Harohalli, Kanakapura Road, Bengaluru South Dt., 562112 India; 4 Manipal Institute of Technology Bengaluru, Manipal Academy of Higher Education, Manipal, Karnataka 576104 India

**Keywords:** Uncertainty in intrusion detection, Neutrosophic algebra, eXplainable artificial intelligence (XAI), Uncertain computing, Intelligent decision support, Multi-criteria optimization, Behaviour analysis, Engineering, Mathematics and computing

## Abstract

Owing to incomplete and inaccurate data, intrusion detection in networks is uncertain. The out-of-order packet arrival uncertainty was not analyzed in the existing works, thereby causing false positives in intrusion detection. So, in this paper, a novel Algebraic Average-based Neutrosophic Logic System (AA-NLS)-based out-of-order uncertainty analysis is proposed. Firstly, the nodes are initialized, and the data is sensed. Then, for intrusion detection, the Network Intrusion Detection System (NIDS) is trained. Here, the data is collected and pre-processed. At the time of pre-processing, Weighted Distance Error Function-based K-Nearest Neighbour (WDEFKNN) is utilized to impute the uncertainty caused by missing values. Further, utilizing the Density-Based Beta Distribution Soergel Spatial Clustering of Applications with Noise (DBBDSSCAN), the behavioural patterns are grouped. Then, the features are extracted, and by utilizing AA-NLS, the epistemic uncertainty caused by out-of-order packet arrival is analyzed**.** In the meantime, by utilizing the Hidden Laplace Witten Bell Markov Model (HLWBMM), the temporal patterns are assessed. Then, the intrusion is detected by the Generalized Riccati Uniform Scaling Orthogonal-based Gated Recurrent Unit (GRUSO-GRU). Here, utilizing the Pareto Entropy-based SHapley Additive exPlanation (PESHAP) model, the explainability of the intrusion detection is carried out. If there is no intrusion, then data transmission is continued. And, if there is intrusion, then the gathered alerts are prioritized and transmitted utilizing the multi-criteria-based Log-Cosh Fennec Fox Optimization Algorithm (LCFFOA). Hence, the intrusion is detected by the proposed framework with an accuracy of 99.299% under the full multi-class setting, demonstrating effective performance compared to prevailing Deep Learning (DL)-based NIDS approaches.

## Introduction

Cybersecurity plays an important part for organizations and individuals in today’s technology development and highly connected digital ecosystem^1^. The network traffic has increased due to the rise in internet-connected devices, Internet of Things (IoT) systems, and other factors, thus making the information system susceptible to cyber-attacks^2^. Therefore, the NIDS acts as a fundamental system for defence against malicious activities, unauthorized access, and so on^3^. The conventional NIDS depended on the signature-based intrusion identification and rule-based methods^4^. Therefore, the emerging threats that depended on predefined patterns could not be identified. For intrusion detection, the prevailing systems used Machine Learning (ML) and DL^5^. For threat detection, most of the prevailing works utilized ML models, such as Support Vector Machine (SVM) and Decision Tree (DT)^6,7^.

The Recurrent Neural Network (RNN) and Long Short-term Memory (LSTM)^8,9^ used to detect intrusions in most of the other conventional works. Though the intrusion was detected accurately, the models were either complex or had a low convergence rate, impacting the intrusion detection’s overall accuracy^10^. Additionally, several uncertainties, such as packet fragmentation, temporal inconsistency, order-of-packet arrival, and so on, were encompassed by the network traffic’s dynamic nature^11^. The uncertainties concerning the missing values in the data were fixed in some of the existing works. Furthermore, the packets that arrived at variable delays and confused the NIDS based on normal and attack classes were not analyzed^12^. The fragmented data, delayed transmission, and other uncertainties were analyzed by only a few models. Therefore, the NIDS that handles the uncertainty is of utmost importance for enhancing cybersecurity concerning data transmission in the network system.

Additionally, several limitations were faced by the prevailing intrusion detection models in improving cybersecurity. Firstly, most of the ML-based models were unable to identify the malicious content that appeared as benign^13^. Then, the temporal dependencies, which were susceptible to concept drift, could not be captured by the DL models efficiently^14^. Further, there occurred a delay in warning the central server concerning the detected intrusion in certain existing works. Delayed threat detection and high false positives were caused by these limitations^15^. Additionally, for accurate threat prediction, it is essential to capture the network states that are not directly observable and the network’s collective behaviour. This paper aims to measure and handle the uncertainties, model the temporal sequence, and incorporate the interpretation of decision-making for an effective intrusion detection mechanism appropriate for modern dynamic networks. So, in this paper, a novel AA-NLS-based epistemic uncertainty analysis and GRUSO-GRU-based intrusion detection for cybersecurity is proposed.

### Problem statement

The existing works related to network intrusion detection had some limitations. The traditional studies failed to handle the problem of out-of-order packet arrival, which was known as the epistemic uncertainty. As a result, the malicious content might be analyzed as normal and vice versa, thus degrading the effectiveness of intrusion detection. Also, the conventional^16^ generated a higher volume of alerts, and the alerts were gathered in queues when this surpassed the processing capacity. This issue created backlogs, and the critical security events were delayed. Likewise, the prevailing^17^ didn’t identify the meaningful patterns and relationships, thereby causing delayed intrusion detection and low situational awareness. Similarly, the uncertainty caused by missing or incomplete values (i.e., the model could not fully know the network state) was not considered^18^, thus leading to improper intrusion detection. Also, the existing^19^ fully relied on the observed features and were unable to capture the sequential dependencies and network behaviour’s hidden dynamics, thereby reducing the detection accuracy. Additionally, most of the prevailing NIDS had lack of insight into why some suspicious events were detected as malicious, thereby degrading the overall model’s transparency and trust.

### Objectives

The proposed framework has many key objectives. For analyzing the out-of-order packet arrival uncertainty that results from network latency, buffering, and queuing in a distributed system, the AA-NLS is introduced, thus improving the effectiveness of intrusion detection. Also, the proposed LCFFOA is employed to prioritize the alerts proficiently during alert accumulation, queuing, and backlog generation, thereby avoiding the backlogs and delays in critical security events. Here, the maximum detection score, minimum latency, and minimum memory usage are considered as the fitness function. Thus, multi-criteria optimization is executed. For grouping the behavioural patterns, the proposed DBBDSSCAN is established, thus effectively identifying the meaningful patterns in the network data and leveraging the patterns’ collective behaviour. Likewise, the proposed WDEFKNN is utilized to impute the missing values, thus correcting the uncertainty caused by the missing or incomplete values in the intrusion detection dataset. For capturing the current event that depends on the previous event and the hidden dynamics in the network behaviour, the proposed HLWBMM is employed. Similarly, the proposed PESHAP is introduced to provide explanations about the intrusion detection outcomes, thus enhancing the model’s transparency and trust.

### Novelty of the research

The proposed model introduces many novelties, including missing value imputation, behavioural pattern grouping, out-of-order packet uncertainty analysis, temporal pattern estimation, intrusion detection, explainability, and alert prioritization. Here, the proposed WDEFKNN is introduced to perform Missing Value Imputation (MVI), thus rectifying the uncertainty in the intrusion detection dataset. Also, the proposed DBBDSSCAN is employed for behavioural pattern grouping, thus recognizing the meaningful patterns in the network data. Likewise, the proposed AA-NLS is utilized to perform out-of-order packet uncertainty analysis, thus improving the intrusion detection outcomes. Similarly, the proposed HLWBMM is used for temporal pattern estimation, thus capturing the temporal hidden dynamics in the network behaviour. Also, the proposed GRUSO-GRU is established to detect intrusions in the network, thus enhancing the accuracy of prediction. Furthermore, the proposed PESHAP is utilized to provide explanations about the detection outcomes, thus improving the trust in the model. Moreover, the proposed LCFFOA is employed to perform alert prioritization, thus avoiding delays in critical security events. Overall, the proposed method reduces the complexity and increases the reliability of NIDS.

The structure of the remaining paper is: The related works are described in Section “Literature survey”, the proposed methodology is explained in Section “Proposed epistemic uncertain computing methodology for intrusion detection”, the performance of the proposed techniques is assessed in Section “Results and discussion”, and the paper is concluded in Section “Conclusion” with future scope.

## Literature survey

Ref.^16^ presented the enhancement of trustworthiness in intrusion detection with uncertainty quantification. Here, the network data was collected. Regarding Out-of-Distribution (OoD), the Multi-Layer Perceptron (MLP) was utilized for determining the uncertainty. After that, for intrusion detection, the Bayesian Neural Network (BNN) was used. By using active learning, the training samples were analyzed effectively. Conversely, the alerts were backlogged, thus delaying the decision-making.

Ref.^17^ introduced probabilistic BNN for intrusion identification. The network traffic data was gathered. After that, the probabilistic BNN was employed to detect the intrusion. Here, by maximizing the evidence lower bound, the model parameters were learned. Next, the moment-centric predictive uncertainty quantification framework was utilized. Hence, the intrusion was determined with probabilistic explanation as well as uncertainty computation. Nevertheless, the model lagged in detecting the relationship between the patterns, thereby reducing the uncertainty estimation.

Ref.^18^ established a dynamic network security analysis utilizing the uncertainty causality graph. Initially, the Dynamic Uncertain Causal Attack Graph (DUCAG) was built between the network events. Then, the uncertainty was computed based on the Causal Chain-centric Risk Probability calculation (CCRP). After that, the attacker behaviours and the potential attack likelihood were effectively estimated by the CCRP under uncertain time-varying circumstances. Nevertheless, the performance of identifying the attack was mitigated by the absence of network information.

Ref.^19^ suggested a soft computing methodology for an Intrusion Detection System (IDS). Primarily, the network packet data was collected as well as pre-processed. After that, the features were selected, and the information gain was determined. Furthermore, to detect the network packets as attacked or normal, the Adaptive Neuro-Fuzzy Inference System (ANFIS) was used. Hence, the intrusion with uncertainty was detected efficiently. Nevertheless, due to the avoidance of capturing the hidden temporal patterns, the detection accuracy was low.

Ref.^20^ developed a Fuzzy Graph Attention Network (FGATN) for intrusion detection. Firstly, duplicate data removal, categorical encoding, and normalization were carried out for the collected network data. Then, the fuzzy sets were set for each traffic flow, and the fuzzy graph was constructed. Later, the edges were pruned and fed to the FGATN for attack identification. Thus, the attack was precisely classified by the FGATN model. But, regarding the reasoning of intrusion detection, the model lacked transparency as well as trust.

Ref.^21^ estimated argumentation-centric query answering with uncertainty in cybersecurity. By using the Defeasible Logic Programming with probabilistic Extension (DeLP3E) model, the knowledge was represented. Then, the data structure was built by utilizing the augmented graph. Further, to enhance the trust of the model, probabilistic reasoning regarding uncertainty and the visualization using XAI were also carried out. Nevertheless, the uncertainty owing to out-of-order packet arrival was not determined, thereby mitigating the uncertainty computation.

Ref.^22^ integrated network anomaly detection utilizing Quantum Neural Network (QNN). Firstly, the network data was gleaned, and the important features were filtered. Then, the encoding tables were assessed and encoded with the chosen features. Finally, using the QNN, the intrusion into the quantum computers was detected accurately. In contrast, the data was not pre-processed, causing the analysis of noisy data and a reduction in anomaly detection.

Ref.^23^ determined the epistemic uncertainty centered on streamlined as well as resource-efficient estimation. Firstly, by using data augmentation, the data distribution was enhanced. Then, the uncertainty distribution was mapped based on the streamlined and resource-efficient estimation. After that, for effectively determining the intrusion with uncertainty, the Auto Encoder (AE) was utilized. But, the slow convergence during the process was not rectified, reducing the model’s total performance.

Ref.^24^ examined intrusion detection in an IoT network utilizing DL. Here, network data was gathered as well as pre-processed. Then, the features were extracted, and the significant features were chosen. Furthermore, for intrusion identification, the Distributed Deep Model (DDM) was utilized. Hence, the intrusion was detected accurately. In contrast, the uncertainty was not analyzed, thereby mitigating the trust in the attained intrusion detection output.

Ref.^25^ deployed the IDS for cybersecurity enhancement. Here, the Trust-centric IDS was designed for the Routing Protocol in a Lower-power-lossy-network (TIDSRPL). For routing the data, the Minimum Rank with Hysteresis Objective Function (MRHOF) was utilized. Then, the malicious activity was identified precisely based on TIDSRPL. Yet, the model was computationally complex and delayed the intrusion detection.

Ref.^26^ analysed XAI-based intrusion detection for attack classification using a DL model. Primarily, the Communications Security Establishment-Canadian Institute for Cybersecurity- Intrusion Detection System 2018 (CSE-CIC-IDS2018) dataset was collected. Then, the data was augmented, and the features were extracted using the EfficientNet module. Further, based on the Multiplicative Luong Attention Deep Residual Multiscale Convolutional Neural Network approach, the network traffic samples were categorized precisely. Finally, the insights of the classified data were improved using SHapely Additive exPlanations (SHAP) and Local Interpretable Model-agnostic Explanations (LIME) models. Yet, the model could not adapt to real-world network conditions.

Ref.^27^ assessed dual branch semi-supervised intrusion detection model based on transformers. A graph was constructed regarding the network data from the CSE-CIC-IDS2018 dataset. Then, the high-order informations were captured. Afterward, the Graph Neural Network (GNN) with Transformer was utilized to capture the contextual semantic information and complement the local behavioral pattern. By using Multi-head Latent Attention (MLA), a transformer encoder, and a graph attention layer, the GNN-Transformer detected the intrusion accurately. However, the emerging attack types were misclassified.

Ref.^28^ established quantum Transfer Learning (TL) for cybersecurity threat detection. At first, the CSE-CIC-IDS2018 dataset was collected. Then, the features were extracted and embedded using a transformer. After that, the quantum TL approach with QNN optimized the parameters and detected the threat effectively. For the malicious class, the alert was generated and sent to the user. On the other hand, the model was computationally complex, delaying the network intrusion detection.

Ref.^29^ conducted DL-enabled intrusion detection system for IoT security. The network traffic data from the CSE-CIC-IDS2018 dataset was collected. Next, the spatial features from the network traffic data were collected using a Convolutional Neural Network (CNN). Afterward, the Gated Recurrent Unit (GRU) effectively learned the temporal dependencies and predicted the intrusion in the network. Thus, the network to be benign or an attack class was determined efficiently. But, the missing values were not imputed, causing an error during network intrusion classification.

Ref.^30^ investigated intrusion detection based on temporal analysis with ML and DL algorithms. Here, the process was carried out regarding the network detection in the application layer. At first, the CSE-CIC-IDS2018 dataset was collected. Then, the Shannon entropy, AutoRegressive Integrated Moving Average (ARIMA), Hölder local exponent, and moving averages models were utilized for determining uncertainty. Finally, the LSTM, which was an advanced RNN approach, was used to detect intrusion in the network. Hence, the attack was classified precisely. On the contrary, the XAI was not applied, reducing the transparency of the model.

## Proposed epistemic uncertain computing methodology for intrusion detection

**Fig. 1:**
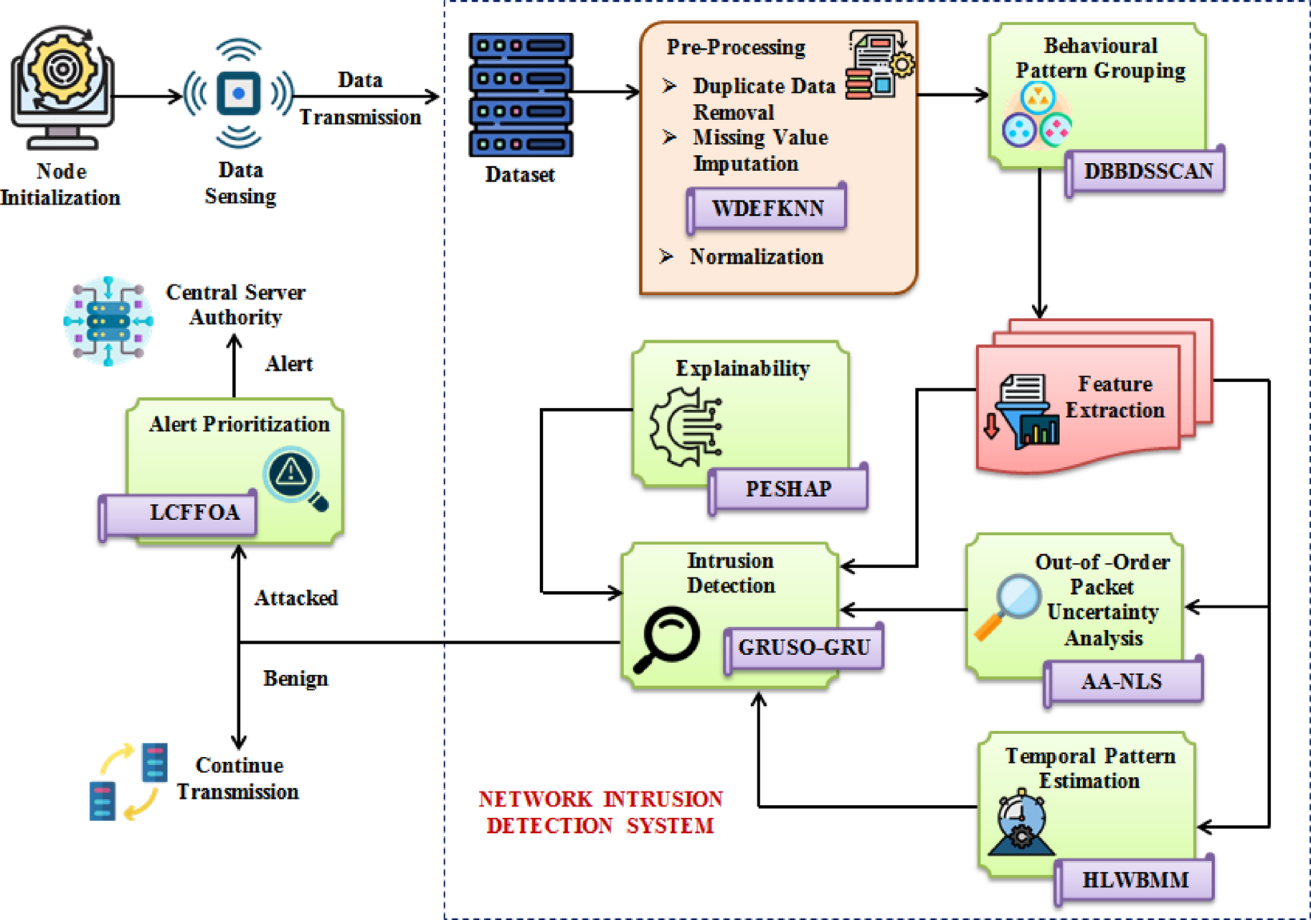
Architecture of the proposed system.

Here, the out-of-order packet arrival uncertainty is analyzed, and the intrusion in the network is identified to enhance cybersecurity. MVI, behavioural pattern grouping, out-of-order packet uncertainty analysis, temporal pattern estimation, intrusion detection, explainability, and alert prioritization are the important steps involved in the proposed system. Figure 1 depicts the proposed work’s structure.

The proposed model demonstrates improved performance by analyzing the out-of-order packet uncertainty and provides an alert by prioritizing them. Here, the missing values present in the dataset are imputed by using the WDEFKNN technique, thus avoiding uncertainties. Afterward, the behavioural patterns are grouped by employing DBBDSSCAN, thus capturing the meaningful behavioural patterns in the network. Then, from the extracted features, the out-of-order packet uncertainty analysis is done by using AA-NLS, thus enhancing the effectiveness of intrusion detection. Thereafter, temporal pattern estimation is carried out by employing HLWBMM, thus obtaining the temporal hidden dynamics in the network. Next, the intrusion is detected based on GRUSO-GRU, thus improving the accuracy. Afterward, the explanations about the prediction outcomes are provided by using PESHAP, thus enhancing the transparency and trust in the model. Eventually, the alerts are sent to the central server authority by prioritizing them using LCFFOA, thus avoiding delays and backlogs. The processes involved in the proposed model are explained briefly below,

### Node initialization & data sensing

The uncertainty computing-based intrusion detection approach begins with the initialization of the network nodes $\left( J \right)$. This $\left( J \right)$ renders the baseline for data sensing and network-based transmission and ensures consistent behaviour for intrusion identification.

1 $$J = \left[ {J^{1} ,J^{2} ,J^{3} ,J^{4} ,....,J^{l} } \right]$$

Here, the number of nodes is signified as $\left( l \right)$. Afterthat, from $\left( J \right)$, the data $\left( M \right)$ is sensed and continues for transmission via the network. Next, the intrusion during data transmission is detected.

### Network intrusion detection system

To identify the threats that occur during the data transmission, the NIDS is trained. During the transmission process, cybercriminals often attack the sensed data and steal important information. Thus, the intrusion that occurred during the data transfer via the network is determined to make decisions. The steps involved in training the NIDS are detailed below,

#### Dataset

Initially, the IDS dataset, which comprises details associated with network data, is collected. For analyzing threat detection, the network data $\left( G \right)$ present in the collected dataset are further employed.

2 $$G = \left\{ {G_{1} ,G_{2} ,G_{3} ,....,G_{h} } \right\}$$

where, the number of $\left( G \right)$ is denoted as $\left( h \right)$. After that, the pre-processing of $\left( G \right)$ is done.

#### Pre-processing

Here, regarding duplicate data removal, MVI, and normalization, $\left( G \right)$ is pre-processed. The pre-processing aids in mitigating noise as well as uncertainty in the input data. Hence, the intrusion detection processing is strengthened.

- Duplicate data removal

Initially, the duplicate data present in the collected data is eliminated. Duplicate data is removed from $\left( G \right)$ as it distorts the learning process of intrusion detection. The duplicated data removal output is notated as $\left( {G^{\prime}} \right)$. After that, the missing values are imputed.

- Missing value imputation

Moreover, by using the WDEFKNN model, the missing values present in $\left( {G^{\prime}} \right)$ are imputed. Regarding the absence of network state data, these missing values cause uncertainty, thus affecting the intrusion detection performance. Here, the K-Nearest Neighbour (KNN) is chosen. KNN imputes missing values based on the most similar instances and preserves the local patterns. Nevertheless, choosing a too small or too large “K” parameter causes poor imputation. Therefore, to estimate the “K” parameter, the Weighted Distance Error Function (WDEF) is used.

Initially, by choosing the “K” parameter $\left( \kappa \right)$ using WDEF, the poor imputation and dilution of local patterns are avoided. This WDEF selects the number of neighbours and prevents including numerous distant points.

3 $$\kappa = \frac{1}{h} \times \sum\limits_{h} {\left[ {G^{\prime}} \right]^{2} * \left( {\frac{1}{1 + b\left( i \right)}} \right)}$$

where, $\left( b \right)$ is the average distance between the neighbouring data, and $\left( i \right)$ is the weight assigned to the neighbouring data. Now, regarding $\left( \kappa \right)$, the missing values are imputed as,

4 $$\dddot G = \frac{{\sum\limits_{h} {\left\langle {i\,\left[ {G^{\prime},\kappa } \right]\,} \right\rangle * G^{\prime}} }}{{\sum\limits_{h} {\left\langle {i\,\left[ {G^{\prime},\kappa } \right]\,} \right\rangle } }}$$

Here, the missing value imputed data is denoted as $\left( {\dddot G} \right)$. Hence, the uncertainty regarding the missing network information is rectified. Finally, $\left( {\dddot G} \right)$ is normalized.

- Normalization

Then, the normalization is done to consider every input on the same scale. Here, by using the min–max technique, $\left( {\dddot G} \right)$ is normalized. The min–max technique rescales all the data into a predefined range of 0 to 1. This ensures fair contribution of all the data utilized for analysis.

5 $$\tilde{G} = \left[ {\dddot G - \dddot G_{\min } } \right]/\left[ {\dddot G_{\max } - \dddot G_{\min } } \right]$$

where, $\left( {\tilde{G}} \right)$ is the normalized data, which is the final pre-processed data, and $\left( {\dddot G_{\min } ,\dddot G_{\max } } \right)$ are the minimum and maximum values of $\left( {\dddot G} \right)$, respectively. Later, the behavioural patterns are clustered.

#### Behavioural pattern grouping

Then, the behavioural pattern grouping is carried out using the DBBDSSCAN technique to identify the meaningful patterns in $\left( {\tilde{G}} \right)$ and leverage the patterns’ collective behaviour. Here, the Density-Based Spatial Clustering of Applications with Noise (DBSCAN) is selected for behavioural pattern grouping. DBSCAN is capable of detecting irregular, sparse, and anomalous behavioural instances within the input data. And, DBSCAN doesn’t require the number of clusters, making it appropriate for grouping behaviours that are dynamic and do not follow a predefined structure. However, DBSCAN is sensitive to the selection of the epsilon and minpts (minimum points) parameters. Thus, using the Soergel Distance (SD), the epsilon parameter is estimated. The minpts are determined using the Beta Distribution (BD) function. Let the input data $\left( {\tilde{G}} \right)$ with $\left( f \right)$ numbers be signified as,

6 $$\tilde{G} \to \left[ {\tilde{G}_{1} ,\tilde{G}_{2} ,\tilde{G}_{3} ,....,\tilde{G}_{f} } \right]$$

Next, by using the SD formula, the epsilon parameter $\left( \varepsilon \right)$, which defines the neighbourhood radius around each data point, is estimated. Here, while accounting for magnitude differences, the SD measures the dissimilarity between feature vectors.

7 $$\varepsilon = \frac{1}{f} * \left[ {\frac{{\sum\limits_{f} {\left| {g_{1} - g_{2} } \right| * \tilde{G}} }}{{\sum\limits_{f} {\max \,\left( {\tilde{G}} \right)} }}} \right]$$

where, $\left( {g_{1} ,g_{2} } \right)$ are the data points in $\left( {\tilde{G}} \right)$. Hence, the maximum distance utilized for grouping the data is assessed. Then, the minpts parameter $\left( \alpha \right)$ is determined using the BD function. $\left( \alpha \right)$ represents the minimum number of points required to form a dense region during grouping. Here, the BD function automatically adapts to data skewness.

8 $$\alpha = \left[ {\delta \left( {g_{1} } \right) + \delta \left( {g_{2} } \right)} \right]/\left[ {\delta \left( {g_{1} ,g_{2} } \right)} \right]$$

where, the Gamma function that generalizes the fractional function in the input data is denoted as $\left( \delta \right)$. Next, based on the data feature, the core point $\left( L \right)$ utilized to group the behavioural pattern is determined after estimating epsilon $\left( \varepsilon \right)$ and minpts $\left( \alpha \right)$.

9 $$L = \tilde{G}\,\left( {\varepsilon ,\alpha } \right)$$

Lastly, the behavioural pattern grouping that analyzes the collective behaviour, meaningful patterns, and their relationships is assessed.

10 $$H = L\left[ {\tilde{G}} \right] * \left( {I \to I^{\max } } \right)$$

where, $\left( H \right)$ is the behavioural pattern grouped output, and $\left( {I,I^{\max } } \right)$ are the present and maximum iterations, respectively. Thus, the grouping continues until all the data points are grouped regarding $\left( {I,I^{\max } } \right)$. Hence, situational awareness is maintained through this grouping, and the decision-making is further enhanced. Later, the features are extracted.

#### Feature extraction

Here, from $\left( H \right)$, the features, such as destination port, protocol, timestamp, flow duration, total forward and backward packets, total bytes exchanged, mean, standard deviation, variance, flow inter-arrival times, forward and backward inter-arrival times, overall flow packets in bytes per second, and so on, are extracted.

11 $$D = \left\langle {D^{1} ,D^{2} ,D^{3} ,D^{4} ,...,D^{m} } \right\rangle$$

where, the extracted features are notated as $\left( D \right)$ and the number of $\left( D \right)$ is implied as $\left( m \right)$. Later, the out-of-order packet uncertainty, which is said to be the epistemic uncertainty, is analyzed.

#### Out-of-order packet uncertainty analysis

Here, by using the AA-NLS, the out-of-order packet arrival in $\left( D \right)$ that occurs due to the network latency, buffering, and queuing in a distributed system is analyzed. This out-of-order packet uncertainty is known as epistemic uncertainty. Owing to the variable delays in packet transmission, epistemic uncertainties are caused by the distance, routing, or congestion in network buffers or queues. These uncertainties arrive at the IDS in a diverse order. Likewise, the packets traveling via numerous paths in a distributed system may arrive at diverse times, leading to temporal misalignment. Here, the Neutrosophic Logic System (NLS) is used for epistemic uncertainty computing. NLS is an extended classic logic system that includes indeterminacy and likelihood values for decision-making. However, if the weighted sum of truth, indeterminacy, and falsity exceeds one, then the uncertainty computing becomes improper. Therefore, during the aggregation of the NLS components, the Algebraic Average (AA) function is used.

Primarily, for the uncertainty computation, the components $\left( \chi \right)$ of the AA-NLS, such as truth $\left( C \right)$ (evidence of attack), indeterminacy $\left( R \right)$ (conflicting uncertainty evidence), and falsity $\left( K \right)$ (evidence of normalcy), are initialized.

12 $$\chi \to \chi \left( {C,R,K} \right)$$

These components are referred as Neutrosophic triplets, which determine the alchemy of uncertainty. Now, regarding the reliability $\left( \phi \right)$ of the input, each data packet in $\left( D \right)$ is recast into a probabilistic tribunal. Therefore, the component $\left( \chi \right)$ values are evaluated as,

13 $$C = \frac{\phi \left( D \right) - \gamma }{{\eta - \gamma }}$$

14 $$R = \left[ {\phi \left( {1 - D} \right) - \gamma } \right]/\left( {\eta - \gamma } \right)$$

15 $$K = {\raise0.7ex\hbox{${\left[ {\left( {1 - \phi } \right) + \phi \left( D \right)} \right] - \gamma }$} \!\mathord{\left/ {\vphantom {{\left[ {\left( {1 - \phi } \right) + \phi \left( D \right)} \right] - \gamma } {\left( {\eta - \gamma } \right)}}}\right.\kern-0pt} \!\lower0.7ex\hbox{${\left( {\eta - \gamma } \right)}$}}$$

where, $\left( {\eta ,\gamma } \right)$ are the upper bound and lower bound values of the normal likelihood in $\left( D \right)$, respectively. Next, the values $\left( C \right)$, $\left( R \right)$, and $\left( K \right)$ are aggregated regarding the AA function to attain the final risk score. This AA function, which reflects the reliability of each component, ensures that the uncertainty-based analysis remains bounded within the range of 0 to 1.

16 $$S = \frac{{\left[ {\left( {n \times C} \right) + \left( {n \times R} \right) + \left( {n \times k} \right)} \right]}}{3 * n}$$

Here, the final out-of-order packet uncertainty computed risk score is represented as $\left( S \right)$ and $\left( n \right)$ denotes the weight that reflects the components’ relative sources. Therefore, the epistemic uncertainty is computed. The pseudo-code for AA-NLS is given below,

**Figure:**
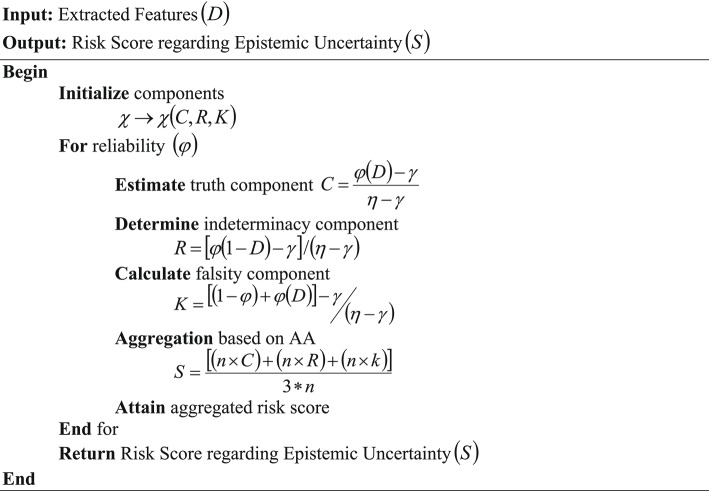
Pseudo-code for AA-NLS.

This $\left( S \right)$ is further subjected to the proposed intrusion detection classifier, and the temporal pattern is analyzed.

#### Temporal pattern estimation

Meanwhile, by using the HLWBMM, the temporal and the sequential dependencies in the network traffic regarding extracted features $\left( D \right)$ are estimated, thus increasing the accuracy of intrusion detection. Here, the Hidden Markov Model (HMM) is chosen for temporal pattern estimation. HMM is a probabilistic method that models the state of network behaviour over time effectively. This HMM assigns each observation to a state based on the sequence of preceding states. Yet, zero probability may occur in HMM, causing improper state assignments. Therefore, the Laplace Witten Bell (LWB) smoothing function is applied in HMM.

Primarily, the extracted features $\left( D \right)$ are fed into the HLWBMM. Later, the parameters $\left( \lambda \right)$, such as forward variable $\left( {V^{1} } \right)$, backward variable $\left( {V^{2} } \right)$, state posterior $\left( {W^{1} } \right)$, and transition posterior $\left( {W^{2} } \right)$, are initialized from the empirical counts of $\left( D \right)$.

17 $$\lambda \to \left( {V^{1} ,V^{2} ,W^{1} ,W^{2} } \right)$$

Here, the probability of the first and the ending observations in the hidden state $\left( y \right)$ at time $\left( t \right)$ is represented as $\left( {V^{1} } \right)$, the probability of future observations is implied as $\left( {V^{2} } \right)$,$\left( {W^{1} } \right)$ is the probability of being in the state $\left( y \right)$ at time $\left( t \right)$, and $\left( {W^{2} } \right)$ notates the probability of being in a state at time $\left( {t,t + 1} \right)$. Now, the parameters $\left( \lambda \right)$ at the given time $\left( t \right)$ are estimated as,

18 $$V^{1} = \left[ {\sum {D * \left( {y,\Im } \right)} } \right]^{\,\,t} \times d$$

19 $$V^{2} = \left[ {\sum {D \times \left( {y,\Im } \right)} } \right]^{\,\,t + 1}$$

20 $$W^{1} = \frac{{\left[ {V^{1} * V^{2} } \right]y\left( t \right)}}{{\sum {\left[ {V^{1} * V^{2} } \right]y\left( t \right)} }}$$

21 $$W^{2} = \frac{{\left[ {V^{1} * V^{2} } \right]y\left( {t + 1} \right)}}{{\sum {\left[ {V^{1} * V^{2} } \right]y\left( {t + 1} \right)} }}$$

22 $$\Im = \left[ {{\raise0.7ex\hbox{${m\left( D \right) + u}$} \!\mathord{\left/ {\vphantom {{m\left( D \right) + u} m}}\right.\kern-0pt} \!\lower0.7ex\hbox{$m$}}} \right] * P$$

where, the transition probability that is evaluated based on the LWB smoothing function is denoted as $\left( \Im \right)$. This LWB smoothing function adds a small constant to all transition counts to prevent zero probability and gives more weightage to the transitions that have not yet been seen. Also, $\left( d \right)$ represents the emission probability, $\left( m \right)$ is the number of $\left( D \right)$, and $\left( {u,P} \right)$ are the constant value and the base distribution regarding global frequency, respectively. Finally, regarding parameters $\left( \lambda \right)$, the temporal pattern $\left( T \right)$ is estimated with $\left( \Im \right)$ as follows,

23 $$T = \sum {\left( {V^{1} ,V^{2} ,W^{1} ,W^{2} } \right)D \times \left[ {\Im ,y\left( t \right)} \right]}$$

Thus, the temporal pattern is estimated, thus capturing the current event depending on previous events and the network states that are not directly observable. Next, the intrusion in the network is detected.

#### Intrusion detection

**Fig. 2:**
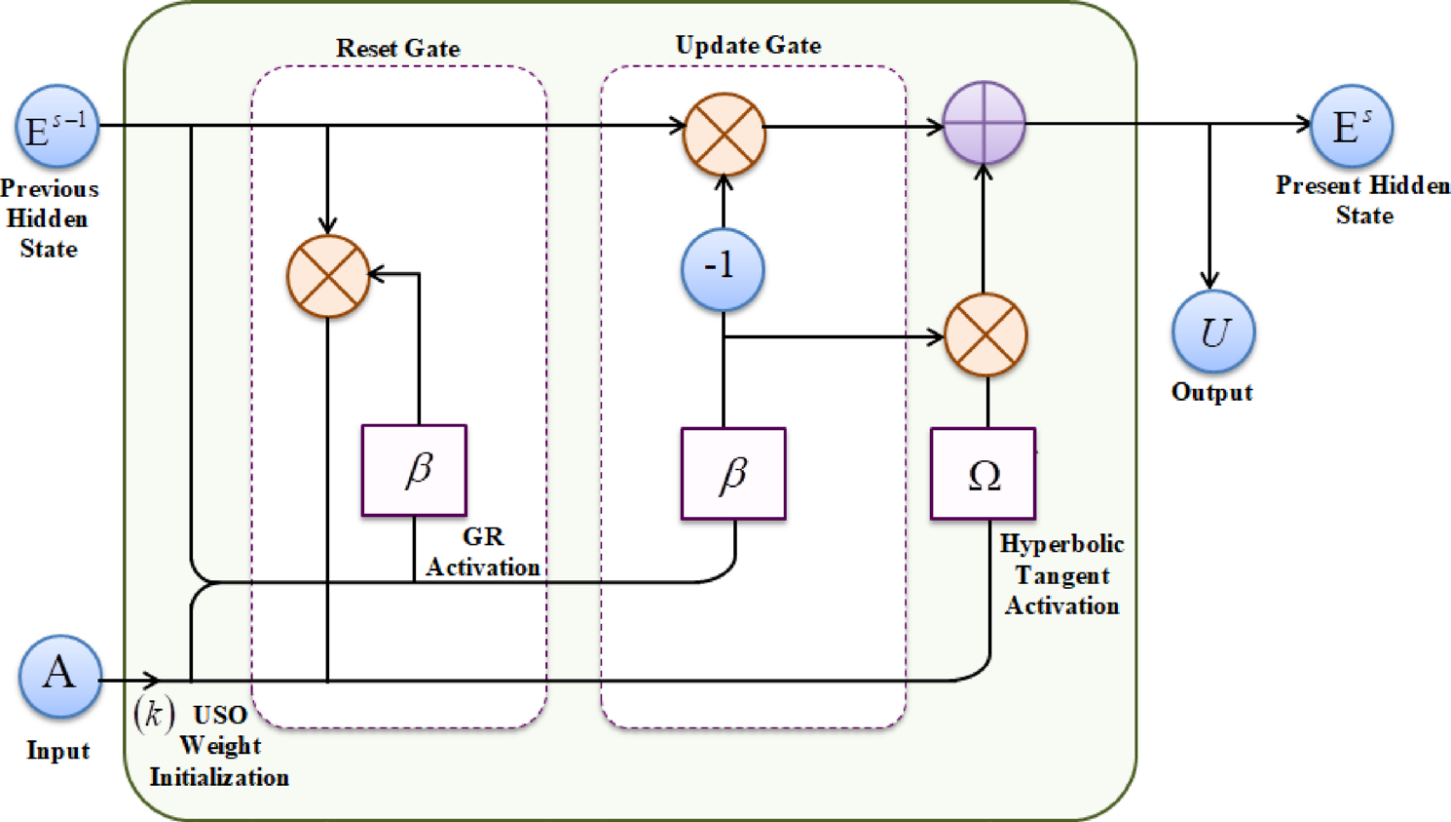
Proposed GRUSO-GRU classifier.

In this phase, regarding the extracted feature $\left( D \right)$, risk score of epistemic uncertainty $\left( S \right)$, and temporal pattern $\left( T \right)$, the intrusion in the network is detected using GRUSO-GRU. The GRU model that effectively analyzes the packet flow is used for intrusion identification. However, the GRU suffers from slower convergence and lower learning efficiency, thus negatively impacting the intrusion detection task. Thus, for overcoming the low learning rate, the Uniform Scaling Orthogonal (USO)-based weight initialization is used. For avoiding slow convergence, Generalized Riccati (GR) activation is used rather than sigmoid activation. Figure 2 illustrates the structural diagram of the proposed GRUSO-GRU classifier.

- Weight initialization

Initially, the inputs $\left( D \right)$, $\left( S \right)$, and $\left( T \right)$ are signified as $\left( A \right)$. During data transmission, this input $\left( A \right)$ is fed into the proposed classifier for threat detection. Now, to overcome the low learning efficiency, the USO is utilized for weight initialization. USO scales the initial weight and balances across hidden and input dimensions.

24 $$k = \sqrt {\frac{2}{w\left( A \right)}} * \left( {\Re ,\dddot k} \right)$$

Here, $\left( k \right)$ is the initialized weight, $\left( w \right)$ is the number of $\left( A \right)$, $\left( \Re \right)$ is the orthogonal matrix, and $\left( {\dddot k} \right)$ is the initial weight of the classifier.

- Reset gate

Furthermore, the data to be retained or discarded is decided in the reset gate. This memory update is carried out centered on the previous hidden state $\left( {E^{s - 1} } \right)$ at time $\left( s \right)$ and the input $\left( A \right)$. Here, using the GR activation $\left( \beta \right)$, the processing is activated. GR activation introduces a tunable parameter $\left( c \right)$ derived from the Riccati differential equation and enables the function to maintain a smooth transition.

25 $$F = \beta * \left\{ {\,k \times \left( {E^{s - 1} ,A} \right) + j\,} \right\}$$

26 $$\beta = {\raise0.7ex\hbox{$A$} \!\mathord{\left/ {\vphantom {A {1 + \left[ {c\,\left( A \right)^{2} } \right]}}}\right.\kern-0pt} \!\lower0.7ex\hbox{${1 + \left[ {c\,\left( A \right)^{2} } \right]}$}}$$

where, the output of the reset gate is notated as $\left( F \right)$ and the bias value is symbolized as $\left( j \right)$.

- Update gate

The update gate is processed after deciding the information that needs to be added or deleted from the classifier’s memory. Here, the previous memory that needs to be retained and the present data that needs to be added to the classifier are decided.

27 $$H = \left\{ {\,1 - \left[ {k \times \left( {E^{s - 1} ,A} \right) + j\,} \right]\, * \beta } \right\} \otimes E^{s - 1}$$

where, the output of the update gate is symbolized as $\left( H \right)$. Afterward, the new hidden state is detected.

- Hidden state

Here, regarding the update gate, input, GR activation, and the hyperbolic tangent action $\left( \Omega \right)$, the present hidden state $\left( {E^{s} } \right)$ of the proposed NIDS classifier is evaluated.

28 $$E^{s} = \left\langle {\left\{ {\Omega * \left[ {k \times \left( A \right) + j} \right]} \right\} \otimes H} \right\rangle \oplus E^{s - 1}$$

29 $$\Omega = \frac{{\exp^{A} - \exp^{ - A} }}{{\exp^{A} + \exp^{ - A} }}$$

Lastly, the threat that occurs in the network during data transmission is identified.

- Output

Thus, based on the update gate and the present hidden state, the intrusion detection output $\left( U \right)$ is estimated. This intrusion is detected in the network during data transmission $\left( M \right)$ that is sensed from a node $\left( J \right)$.

30 $$U = E^{s} \oplus H\,\,\,\,\,\forall \left( {U \to U^{1} /U^{2} /U^{3} /U^{4} /U^{5} /U^{6} /U^{7} } \right)$$

where, $\left( {U^{1} ,U^{2} ,U^{3} ,U^{4} ,U^{5} ,U^{6} ,U^{7} } \right)$ are the benign (normal), Distributed Denial of Service (DDoS), Denial of Service (DoS), brute force, botnet, infiltration, and web attack classes, respectively. The pseudo-code for GRUSO-GRU is given below,

**Figure:**
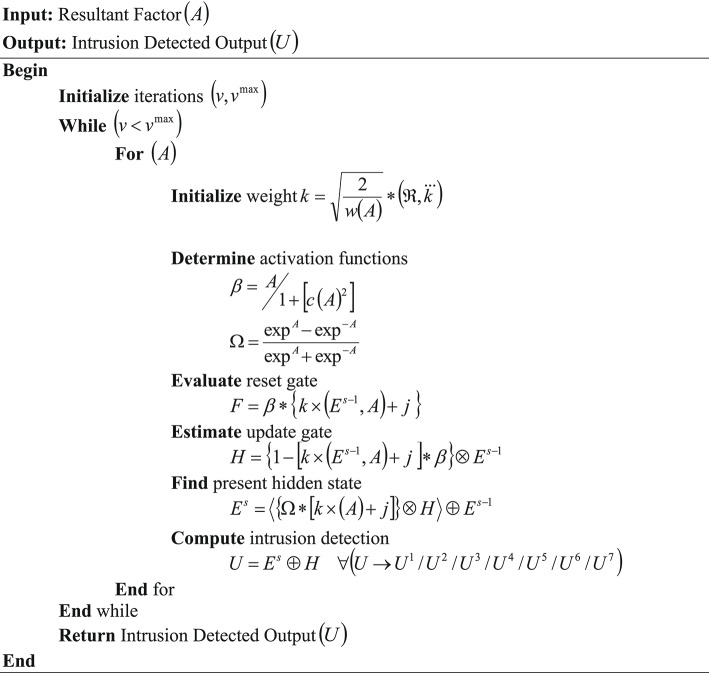
Pseudo-code for GRUSO-GRU.

After training the NIDS, the data $\left( M \right)$ is transferred via the network, and the intrusion is detected. If $\left( {U^{1} } \right)$ is attained, then the data $\left( M \right)$ transmission from the node is continued; if $\left( {U^{2} ,U^{3} ,U^{4} ,U^{5} ,U^{6} ,U^{7} } \right)$ occurs, then the transmission is blocked and the alert is prioritized. Then, the explainability is provided to enhance the transparency of $\left( U \right)$.

#### Explainability

Thereafter, the PESHAP is applied to enhance the explainability regarding intrusion detection. By providing this explainability, the model’s transparency and trust are increased. Here, for providing explainability, the SHAP model that provides insights into how input features influence the intrusion prediction is chosen. However, the underlying structure of the data may not be captured by the kernel function used for assigning the weight, causing suboptimal results. Hence, the Pareto Entropy (PE) is applied to the input of SHAP.

Primarily, $\left( U \right)$ is fed into the PESHAP model. Then, the PE is utilized to estimate the kernel function $\left( B \right)$. PE quantifies the information content of skewed distributions and makes the input more stable.

31 $$B = 1 + \left( {\frac{1}{\mu }} \right) + \left[ {\ln \left( {\frac{U}{\mu }} \right)} \right]$$

where, the scaling parameter is denoted as $\left( \mu \right)$. Next, the explainability $\left( Y \right)$ that provides the transparency in attaining the required intrusion detection is evaluated based on the input $\left( U \right)$, the subset $\left( Q \right)$ of $\left( U \right)$, and the kernel function $\left( B \right)$.

32 $$Y = \frac{{\sum {\left| Q \right|\,! * \left\{ {\,\left| {U\left( B \right)\,} \right| - \left| Q \right| - 1} \right\}\,!} }}{{\sum {\,\left| {U\left( B \right)\,} \right|\,!} }}$$

Thus, the actionable reasoning that provides the transparency and trust of the overall proposed classifier model is achieved. Next, the alert is prioritized for the attained $\left( {U^{2} ,U^{3} ,U^{4} ,U^{5} ,U^{6} ,U^{7} } \right)$.

### Alert prioritization

Finally, the LCFFOA is used to prioritize the alert with multi-criteria optimization, thus avoiding delays in critical security events. Here, the Fennec Fox Optimization Algorithm (FFOA) is chosen for alert prioritization. FFOA adapts the Fennec Fox’s survival skills in the harsh desert environment. Here, the multi-criteria, such as maximum detection score $\left( r \right)$ from $\left( U \right)$, minimum latency $\left( p \right)$, and minimum memory usage $\left( q \right)$, are set as fitness for effective alert prioritization. However, in the exploration phase, the FFOA might generate a suboptimal solution, causing ineffective alert prioritization. Thus, to enhance the exploration phase of FFOA, the Log-Cosh (LC) function is used.

- Position initialization

A high volume of alerts $\left( N \right)$ is generated as the intrusions, such as $\left( {U^{2} ,U^{3} ,U^{4} ,U^{5} ,U^{6} ,U^{7} } \right)$, are detected with XAI $\left( Y \right)$. Therefore, the alerts are prioritized to avoid the delay in alerting the central server authority and to avoid the backlog alert generation. Initially, the position $\left( {N^{x} } \right)$ of the fennec fox (generated alerts)$\left( N \right)$ is initialized.

33 $$N^{x} = N\left\{ {a * \left( {ub^{N} - lb^{N} } \right) + lb^{N} } \right\}$$

where, $\left( {lb^{N} ,ub^{N} } \right)$ are the lower bound and upper bound values of the fennec fox, respectively, and $\left( a \right)$ is the constant value. Then, the fitness function is assessed.

- Fitness function

Now, the fitness function $\left( Z \right)$ is determined to determine the multi-criteria-based optimal solution. Here, for multi-criteria-based fitness estimation, detection score $\left( r \right)$, latency $\left( p \right)$, and memory usage $\left( q \right)$ are utilized.

34 $$Z = \max \left[ {r\left( U \right)} \right] + \min \left( p \right) + \min \left( q \right)$$

This $\left( Z \right)$ is deployed for the fennec fox position update. Next, the position $\left( {N^{x} } \right)$ is updated to determine the optimal solution.

- Position update

Here, the position update is carried out centered on two stages, namely the exploration phase and the exploitation phase.

#### Exploration phase

Initially, the fennec fox detects the prey by utilizing its large ears. As the prey is detected by the fennec fox, it captures the prey and burrows it inside the sand. Here, the LC function is utilized to capture the prey movements beneath the sand for avoiding the suboptimal results during the search. This LC function averts saturation and enhances the sensitivity to subtle variations.

35 $$O = \log \left[ {\frac{{\exp^{{N^{x} }} - \exp^{{ - N^{x} }} }}{2}} \right]$$

where, $\left( O \right)$ is the selected prey movement and $\left( {\exp } \right)$ is the exponential factor. Hence, regarding $\left( O \right)$ and the initial position, the fennec fox’s new position $\left( {N^{x + 1} } \right)$ is updated as,

36 $$N^{x + 1} = N^{x} + \left[ {N^{x} - O\,\left( {N^{x} - 1} \right)} \right]$$

After updating the position in the global search, the fennec fox’s position in the local search is detected.

#### Exploitation phase

As the prey is captured, the fennec fox preserves the food and then escapes from its predators. This strategy is deployed for the position updation $\left( {N^{x + 2} } \right)$ in the exploitation phase.

37 $$N^{x + 2} = N^{x + 1} + \left[ {\frac{{\left( {ub^{N} - lb^{N} } \right) * O\,\left( {N^{x + 1} } \right)}}{\varsigma }} \right]$$

where, the present iteration is notated as $\left( \varsigma \right)$. Now, the multi-criteria optimization, which is the prioritized alert, is attained regarding the fitness function $\left( Z \right)$. Let the prioritized alert be notated as $\left( {N^{best} } \right)$. Hence, regarding $\left( {N^{best} } \right)$, the alerts are sent to the central server authority. Hence, the uncertainty during intrusion detection is effectively computed by the proposed system, thereby enhancing cybersecurity during data transmission in the network. The proposed framework’s performance validation is explained below.

## Results and discussion

In this phase, the proposed system’s performance is assessed and contrasted with the prevailing studies. The metric values are acquired by working in the PYTHON platform.

### Experimental setup

To ensure transparency and reproducibility of the proposed system, the experimental protocol adpoted are explained below.

- Hardware and software details

The hardware requirements of the proposed work are described below,

- *Processor* Intel i5/core i7
- *CPU Speed* 3.20 GHz
- *RAM* 16 GB
- *Hard Disk Space* 32-bit OS
- *Display* 800 × 600

Also, the proposed model is implemented in the working platform of PYTHON. Normally, PYTHON is a high-level, object-oriented, and general-purpose programming language, which is widely used due to its simple and readable syntax. Python code is executed line by line, thereby speeding up the process.

(b) Dataset description

The NIDS is trained for intrusion detection during data transmission. In this, for training and testing the proposed GRUSO-GRU classifier for intrusion detection, the “CSE-CIC-IDS2018”, which is publicly available, is used. This dataset consists of labeled network traffic data collected from numerous days of traffic captures. The CSE-CIC-IDS2018 dataset consists of seven classes, such as benign (normal), DDoS, DoS, brute force, botnet, infiltration, and web attack classes. In the CSE-CIC-IDS2018 dataset, a total of 1,252,835 data are available. The total data is partitioned into 80% and 20%, which are utilized for training and testing purposes, correspondingly. This splitting is conducted using stratified sampling to preserve the class distribution across the training and the testing sets. All primary results are reported under the seven-class setting of the CSE-CIC-IDS2018 dataset. Also, the proposed model employs “Network Intrusion Detection Dataset” and “Canadian Institute for Cybersecurity-Intrusion Detection System 2017 (CIC-IDS-2017)” for cross-dataset generalization. The “Network Intrusion Detection Dataset” consists of 47,736 number of data. Likewise, the “CIC-IDS-2017” dataset contains 2,401,124 number of data. The link for the gathered dataset is rendered in the reference section.

### Performance validation

In this phase, for the CSE-CIC-IDS2018 dataset, the performance validation of the proposed methods, like AA-NLS, GRUSO-GRU, LCFFOA, PESHAP, DBBDSSCAN, HLWBMM, and WDEFKNN, is compared against the traditional baseline methods. The performance metrics are reported as macro-averaged values to ensure a balanced evaluation across all classes.

**Fig. 3:**
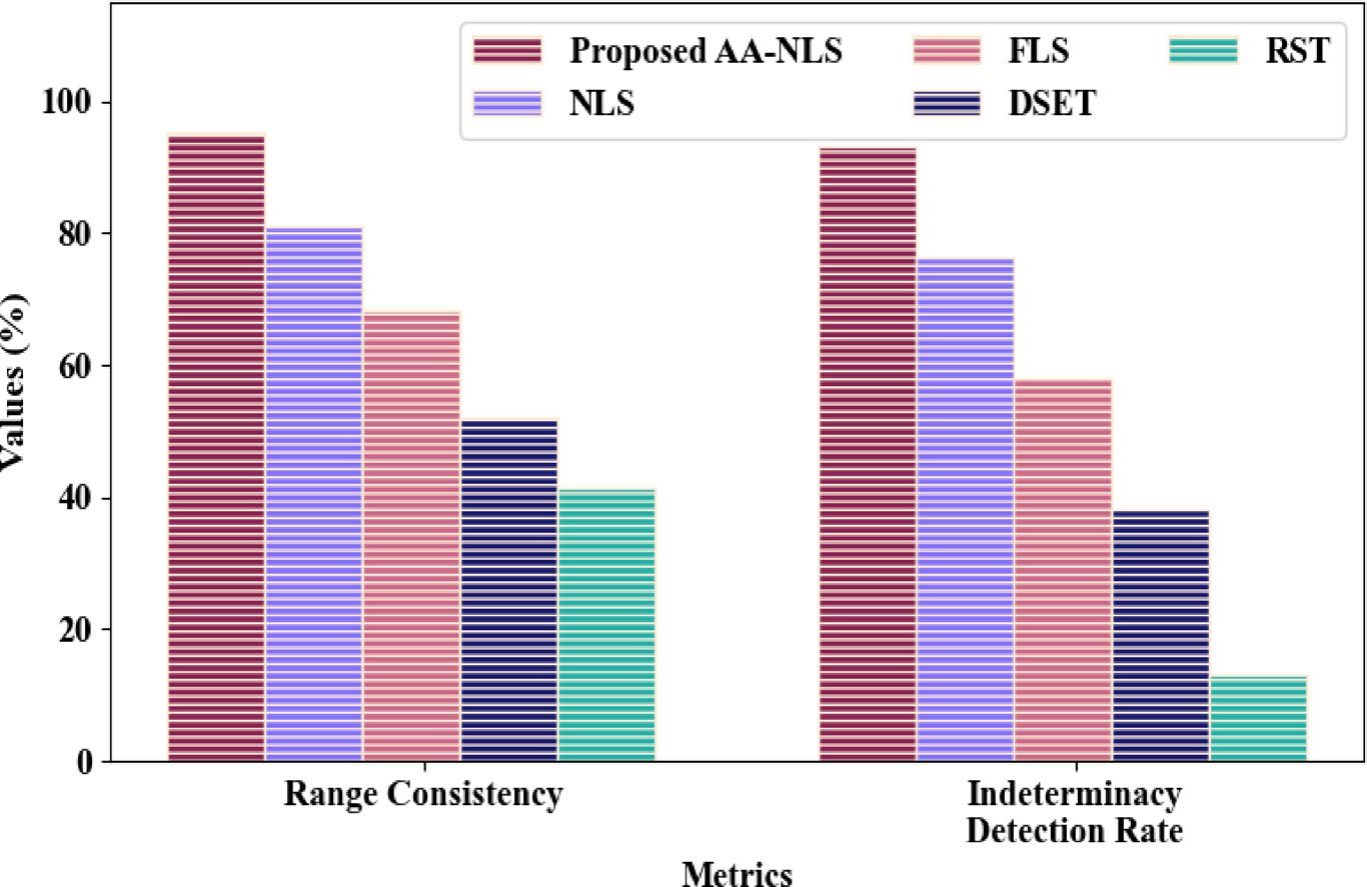
Comparative analysis of AA-NLS.

Figure 3 depicts the proposed AA-NLS and the traditional methods’ comparison concerning out-of-order packet arrival uncertainty (epistemic uncertainty) analysis. The epistemic uncertainty was calculated by the proposed AA-NLS and the existing NLS, Fuzzy Logic System (FLS), Dempster-Shafer Evidence Theory (DSET), and Rough Set Theory (RST) with a range consistency of 95.112%, 80.947%, 68.342%, 52.184%, and 41.4735, and Indeterminacy Detection Rate (IDR) of 93.249%, 76.382%, 57.839%, 38.294%, and 13.115%, correspondingly. When compared to the existing models, the proposed AA-NLS’s performance was enhanced by the summation of the truth, indeterminacy, and falsity components utilizing AA.

**Fig. 4:**
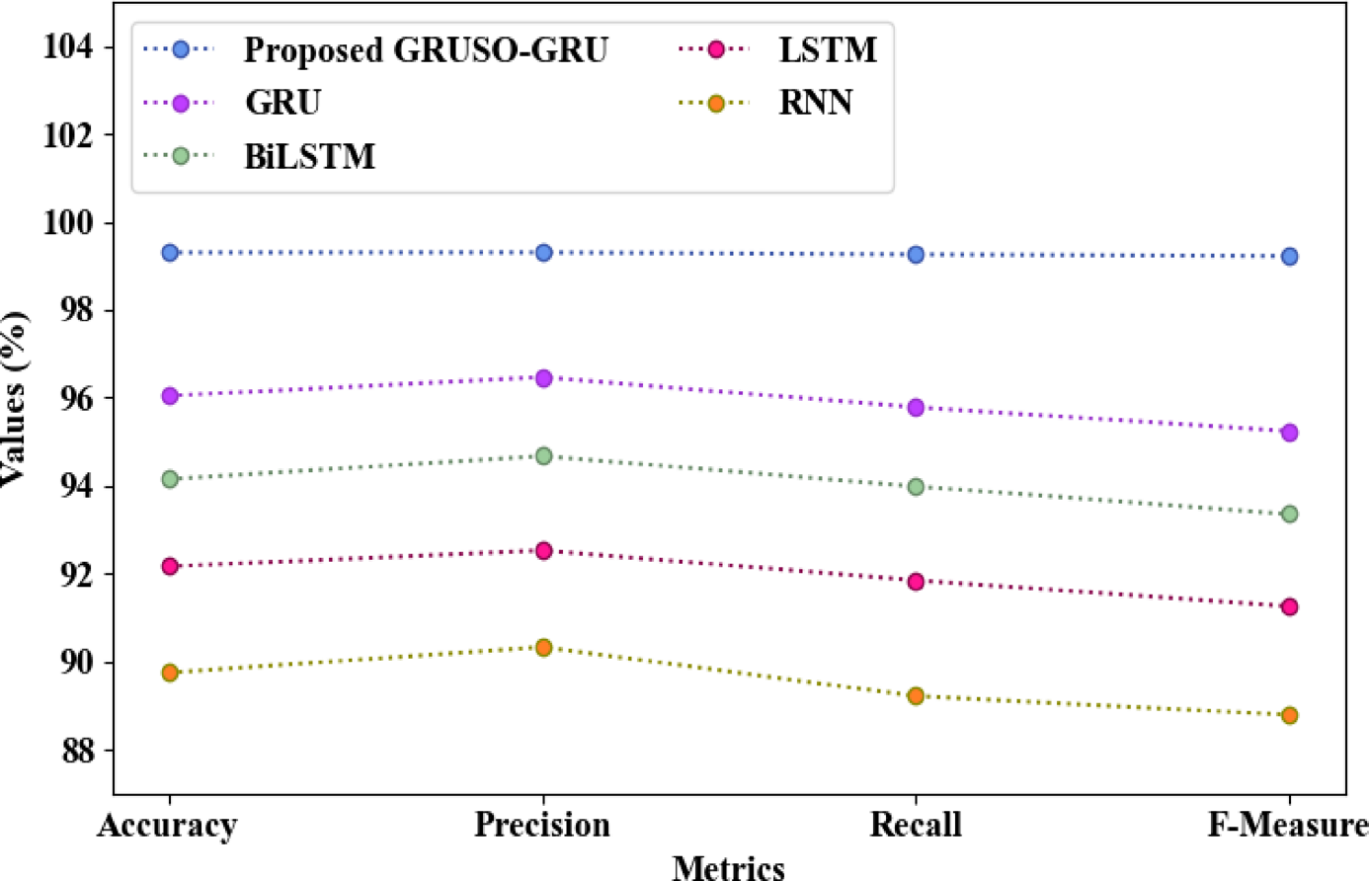
Graphical comparison of GRUSO-GRU.

The comparison of the proposed and the conventional classifiers concerning intrusion detection is depicted in Table 1 and Fig. 4. The threat was identified by the proposed GRUSO-GRU with an accuracy, precision, F-Measure, recall, False Positive Rate (FPR), and False Negative Rate (FNR) of 99.299%, 99.301%, 99.219%, 99.256%, 0.6328, and 0.8842, respectively. Yet, an average accuracy, precision, recall, F-Measure, FPR, and FNR of 93.024%, 93.499%, 92.708%, 92.159%, 4.1566, and 7.152 were achieved by the traditional GRU, Bi-directional Long Short Term Memory (BiLSTM), LSTM, and RNN, respectively. The proposed GRUSO-GRU improved the performance over traditional classifiers, as the low learning efficiency was avoided by utilizing USO and the slow convergence was prevented by utilizing GR activation.

**Table 1:** Comparative analysis of GRUSO-GRU regarding seven-class (multi-class) intrusion detection performance.

Techniques	Accuracy (%)	Precision (%)	Recall (%)	F-Measure (%)	FPR	FNR
Proposed GRUSO-GRU	99.299	99.301	99.256	99.219	0.6328	0.8842
GRU	96.045	96.467	95.784	95.238	1.8945	3.8421
BiLSTM	94.146	94.674	93.984	93.352	3.3742	6.1456
LSTM	92.163	92.526	91.847	91.258	4.7844	8.3821
RNN	89.743	90.327	89.215	88.787	6.5732	10.2384

**Fig. 5:**
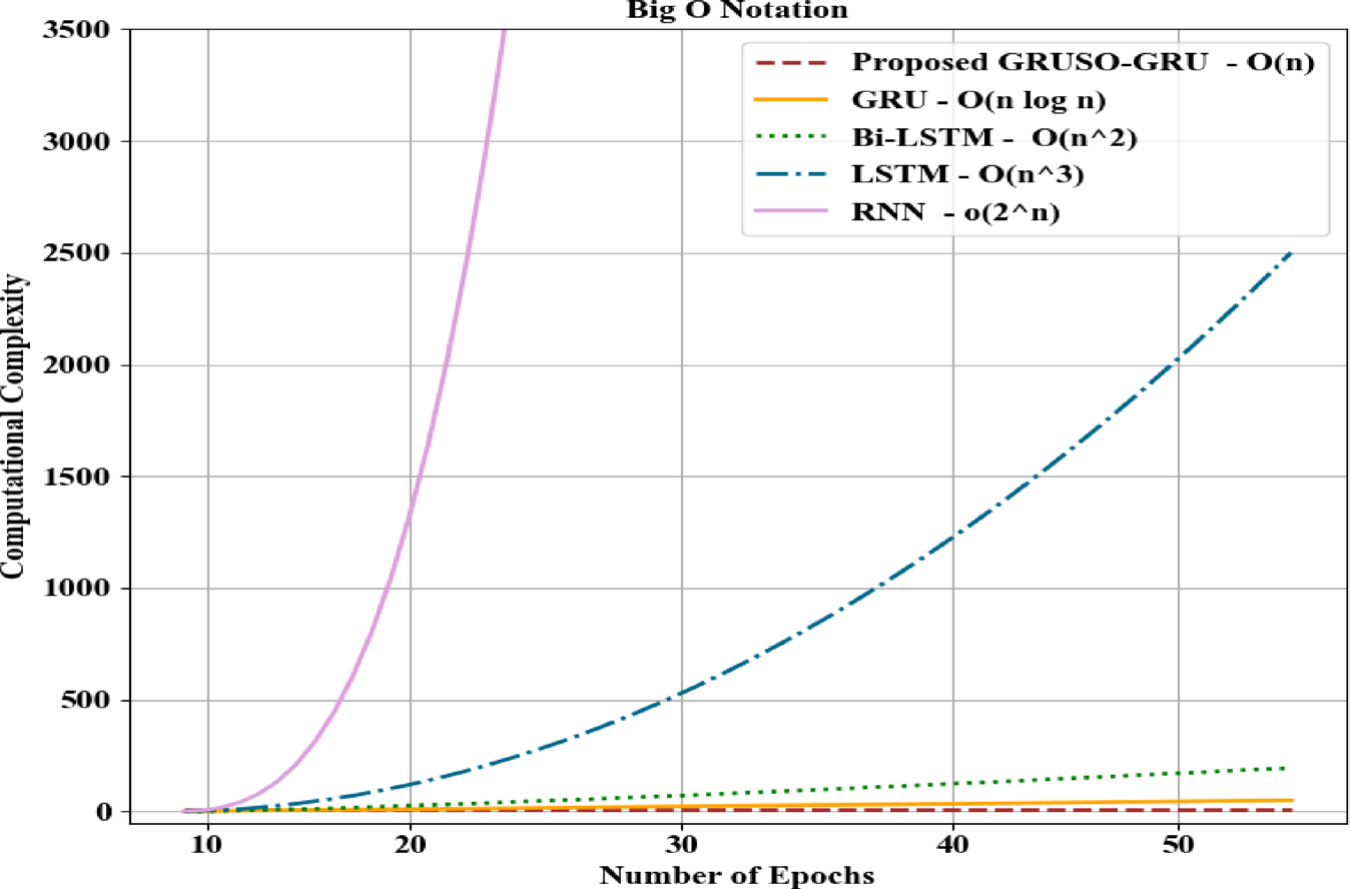
Computational complexity analysis for proposed GRUSO-GRU.

Figure 5 depicts the computational complexity analysis of the proposed GRUSO-GRU and prevailing techniques in terms of Big O notation. Here, the proposed model had low computational complexity owing to the inclusion of USO for elevating the learning efficiency and GR activation for avoiding the slow convergence. In general, Big O Notation indicates the efficiency of an algorithm by displaying how its performance scales with input size. The proposed GRUSO-GRU achieved a low computational complexity of O(n), whereas the prevailing techniques, such as GRU, BiLSTM, LSTM, and RNN, attained a low computational complexity of O(n log n), O(n^2), O(n^3), and O(2^n), respectively. Thus, the effectiveness of the proposed model is proven.

**Fig. 6:**
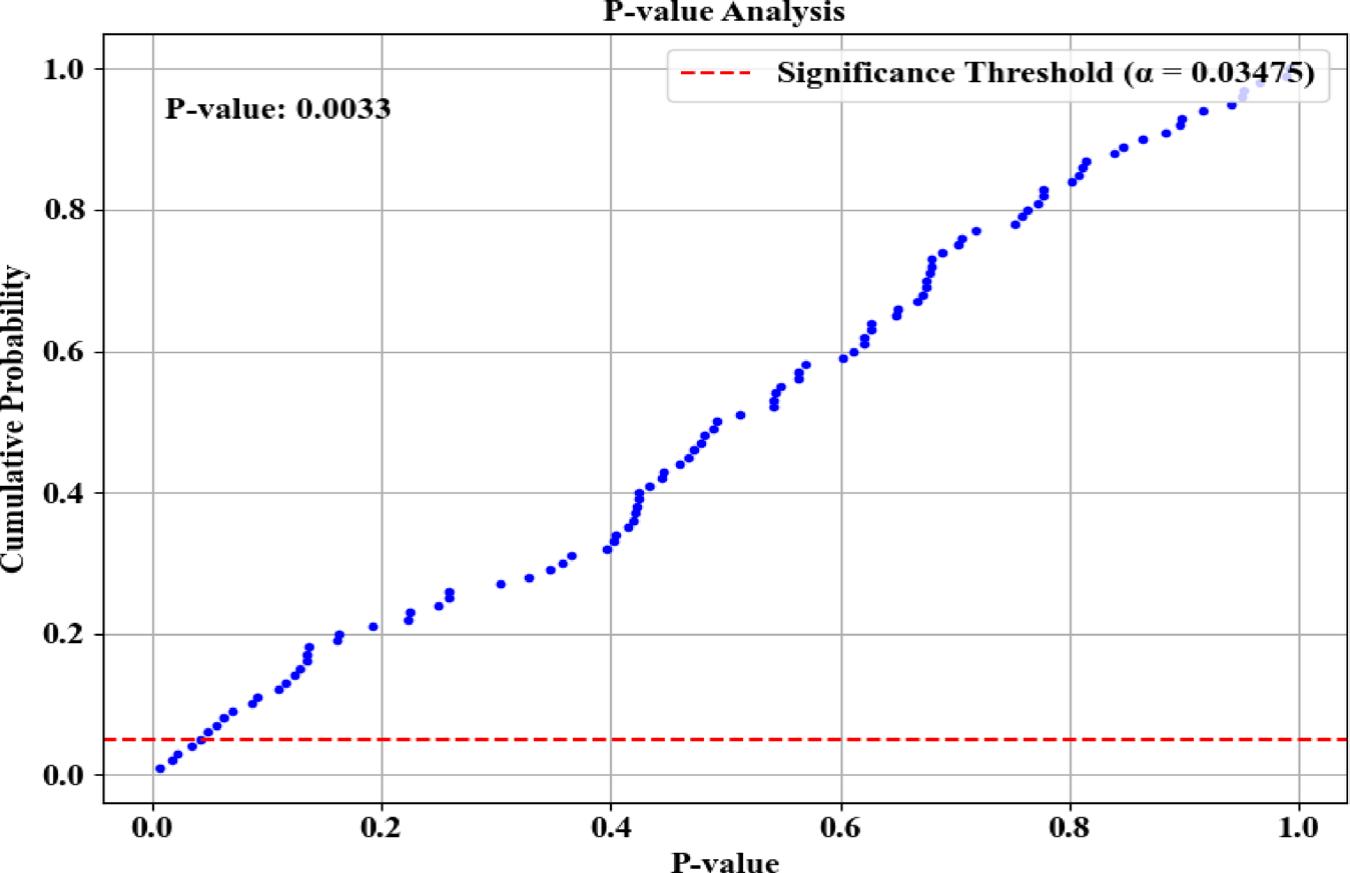
Statistical significance test regarding *P*-value.

Statistical significance test analysis of the proposed model in terms of *P*-Value is provided in Fig. 6. Here, the proposed model had a *P*-Value of 0.0033, which specified that the observed differences were very low based on the random variation. The figure reveals that the input features are not random and are different according to the baseline. Thus, the obtained *P*-Value indicated the proposed model’s improvement in network intrusion detection.

Table 2 depicts the ablation study analysis to specify each module’s contribution. Here, the proposed model with the integration of each module achieved a high accuracy, precision, and recall of 99.299%, 99.301%, and 99.256%, respectively, thus accurately classifying the intrusions. This was because the WDEFKNN handled missing values, DBBDSSCAN captured collective behavioral patterns, AA-NLS addressed epistemic uncertainty due to packet reordering, HLWBMM captured hidden temporal dependencies, and GRUSO-GRU improved learning stability and convergence during intrusion detection. However, the proposed model without Out-of-order packet uncertainty analysis attained a low accuracy (95.781%), precision (95.824%), and recall (95.723%), thus reducing the ability of intrusion detection. Also, the proposed model without behavioural pattern grouping obtained a low accuracy, precision, and recall of 93.109%, 93.289%, and 93.031%, correspondingly, thus identifying meaningful patterns in the network data. Likewise, the proposed model without temporal pattern estimation attained a low accuracy (89.679%), precision (89.756%), and recall (89.534%), thus missing the temporal information. Similarly, the proposed model without missing value imputation attained a very high accuracy of 84.982%, precision of 84.867%, and recall of 84.723%, thus rectifying the uncertainty caused by the missing and incomplete values in the intrusion detection dataset. A cumulative performance degradation was observed when individual phases were removed from the proposed model. Thus, the proposed model, with the integration of each module, such as WDEFKNN, DBBDSSCAN, AA-NLS, HLWBMM, and GRUSO-GRU, obtained enhanced performance in intrusion detection.

**Table 2:** Ablation study analysis.

Techniques	Accuracy (%)	Precision (%)	Recall (%)
Proposed model	99.299	99.301	99.256
Proposed model without out-of-order packet uncertainty analysis	95.781	95.824	95.723
Proposed model without behavioural pattern grouping	93.109	93.289	93.031
Proposed model without temporal pattern estimation	89.679	89.756	89.534
Proposed model without missing value imputation	84.982	84.867	84.723

By utilizing multi-criteria optimization, the alerts concerning the identified intrusion were prioritized in the proposed LCFFOA. Furthermore, the suboptimal results were overcome by the proposed model utilizing the LC function. Table 3 depicts that the proposed model achieved throughput values of 603kbps, 664kbps, 731kbps, 818kbps, and 905kbps and latencies of 2289 ms, 3372 ms, 4382 ms, 5437 ms, and 6289 ms for 100, 200, 300, 400, and 500 nodes, respectively. Conversely, the alerts were prioritized by the prevailing FFOA, Hummingbird Optimization Algorithm (HOA), Mountain Gazelle Optimization Algorithm (MGOA), and African Vulture Optimization Algorithm (AVOA) with average throughput values of 342kbps, 411kbps, 479kbps, 551kbps, and 618kbps and average latencies of 7254 ms, 8315 ms, 9425 ms, 10557 ms, and 11570 ms for 100, 200, 300, 400, and 500 nodes, respectively. It was proven that when analogized with other extant models, the proposed LCFFOA performed better concerning alert prioritization.

**Table 3:** Comparative analysis of LCFFOA.

Methods	Metrics	Number of Nodes				
		100	200	300	400	500
Proposed LCFFOA	Throughput (kbps)	603	664	731	818	905
	Latency (ms)	2289	3372	4382	5437	6289
FFOA	Throughput (kbps)	428	493	563	642	719
	Latency (ms)	4281	5329	6283	7392	8434
HOA	Throughput (kbps)	378	448	517	583	653
	Latency (ms)	6381	7284	8321	9481	10,432
MGOA	Throughput (kbps)	346	417	484	558	606
	Latency (ms)	8219	9183	10,421	11,573	12,732
AVOA	Throughput (kbps)	219	285	352	421	493
	Latency (ms)	10,135	11,462	12,673	13,782	14,682

**Fig. 7:**
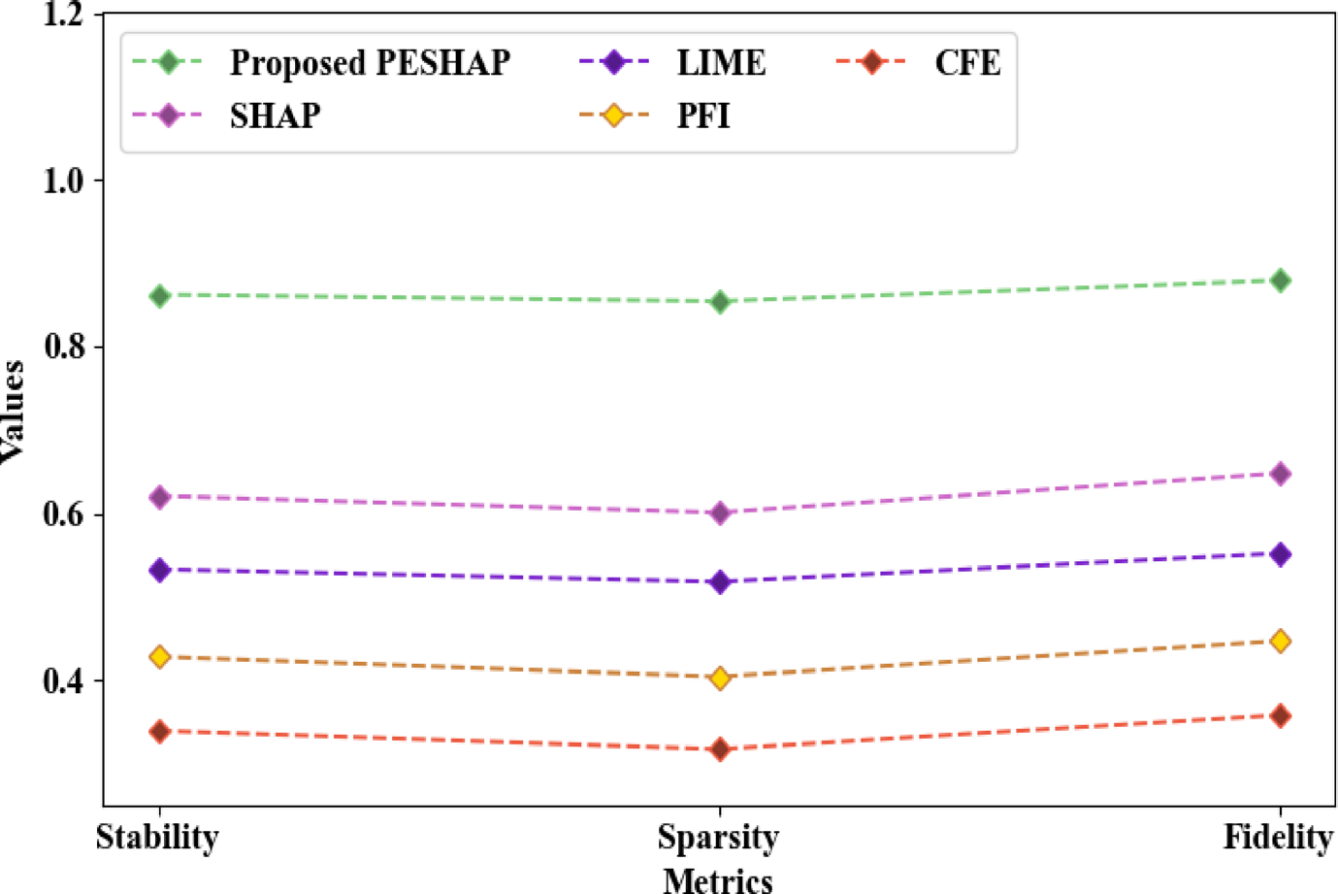
Comparative analysis regarding XAI.

According to Fig. 7, the transparency of the intrusion detection output was enhanced by the proposed PESHAP, with a stability, sparsity, and fidelity of 0.862, 0.854, and 0.879, respectively. However, the conventional SHAP, LIME, Permutation Feature Importance (PFI), and Counter-Factual Explanation (CFE) rendered explainability with a stability of 0.621, 0.533, 0.428, and 0.339, sparsity of 0.601, 0.518, 0.404, and 0.317, and fidelity of 0.648, 0.552, 0.447, and 0.358, correspondingly. Therefore, when contrasted with the traditional methods, the PE’s utilization for capturing the input data’s underlying structure improved the proposed XAI model’s performance.

**Fig. 8:**
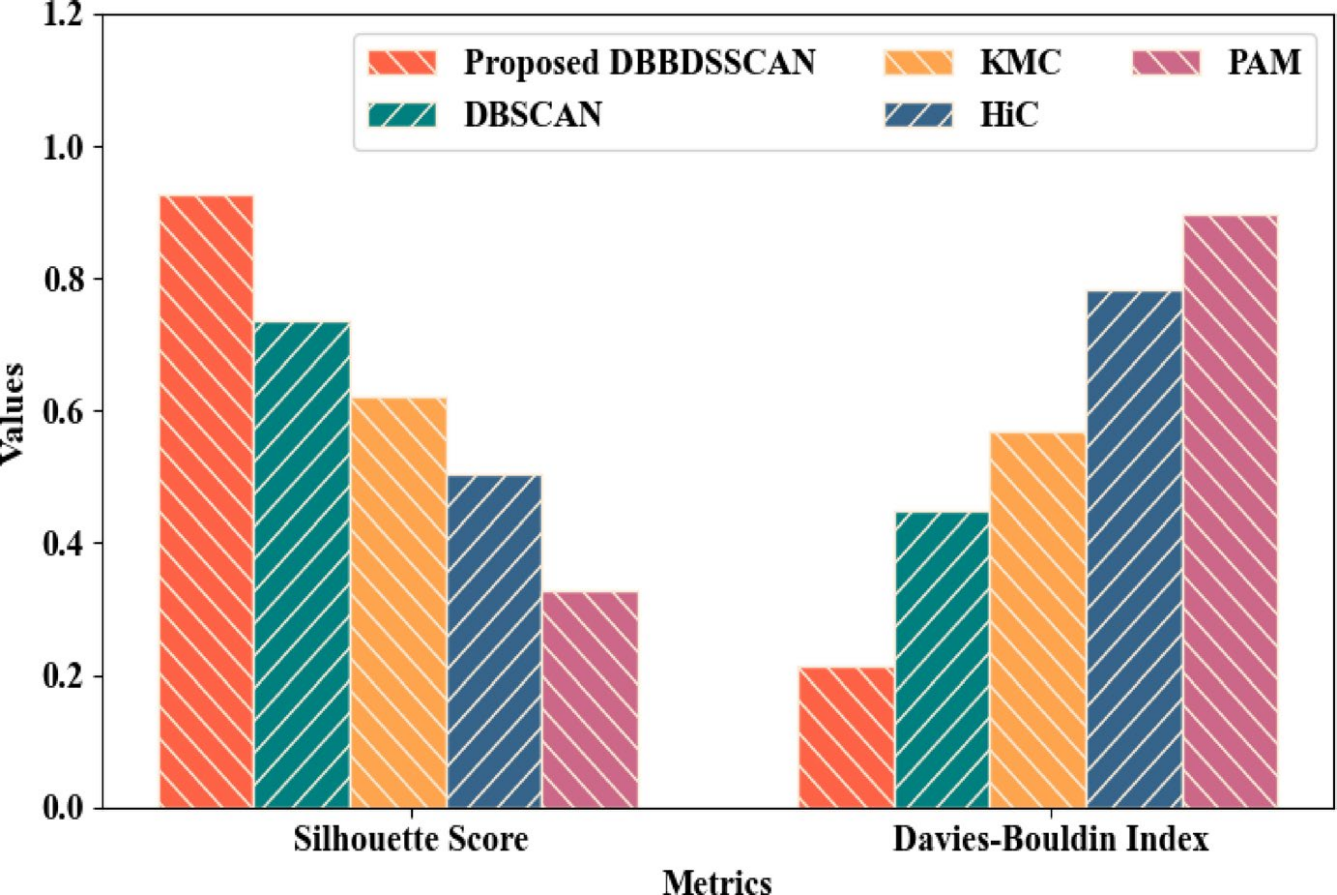
Graphical comparison of DBBDSSCAN.

The SD-based epsilon parameter and the BD-based minpts were utilized for grouping the behavioural patterns existing in the network data. Figure 8 demonstrated that the behavioural patterns were clustered by the proposed DBBDSSCAN with a Silhouette score and Davies-Bouldin-Index (DBI) of 0.928 and 0.214, respectively. Conversely, the existing DBSCAN, K-Means Clustering (KMC), Hierarchical Clustering (HiC), and Partition Around Medoid (PAM) clustered the data with a Silhouette score of 0.735, 0.621, 0.503, and 0.327, and DBI of 0.448, 0.569, 0.783, and 0.897, correspondingly. This depicted that the proposed clustering technique clustered the behavioural patterns better when compared to other conventional clustering models.

**Fig. 9:**
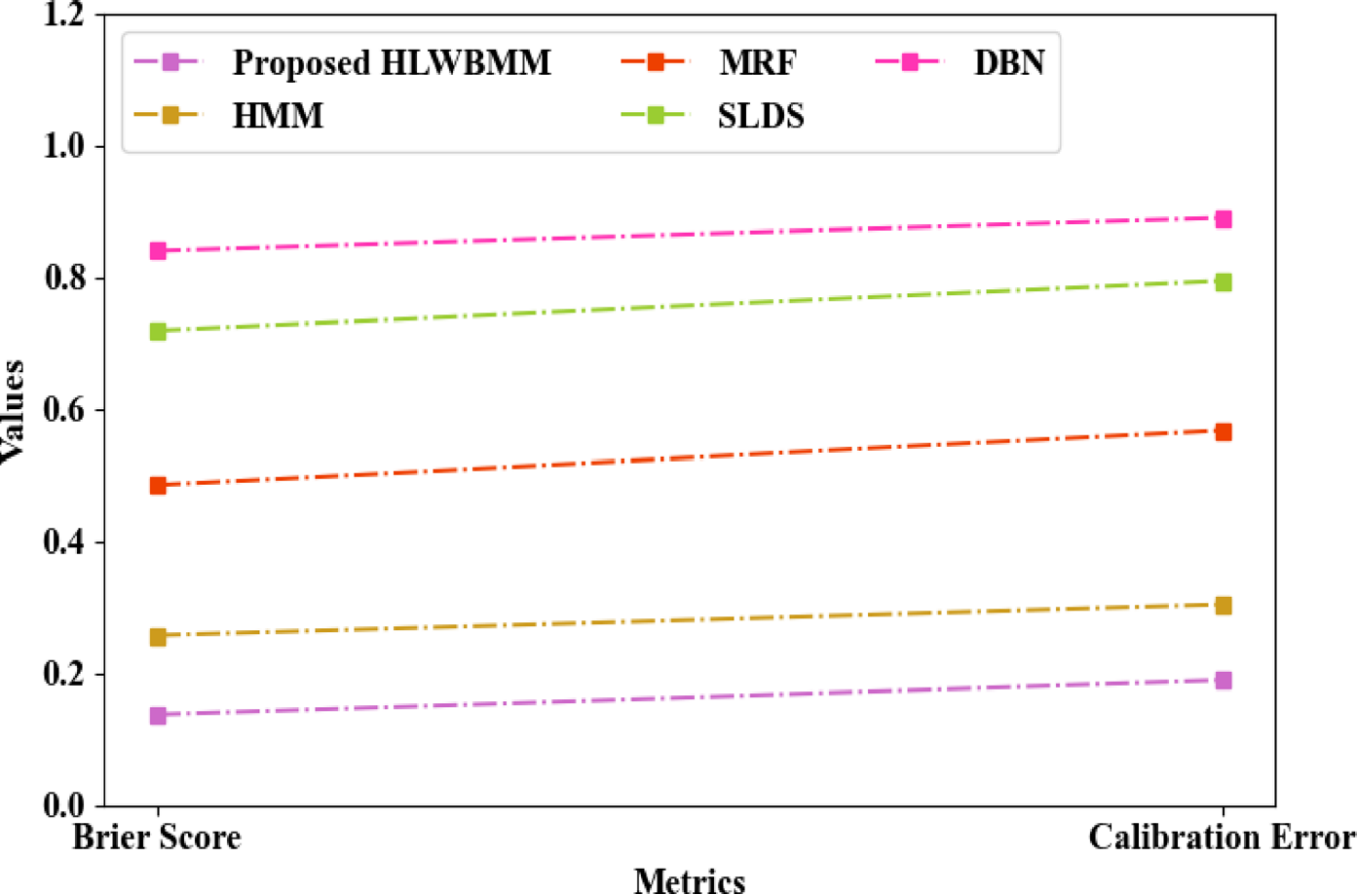
Comparative analysis regarding temporal pattern estimation.

The comparison of the proposed HLWBMM and the extant HMM, Markov Random Field (MRF), Switching Linear Dynamical System (SLDS), and Dynamic Bayesian Network (DBN) concerning temporal pattern estimation is shown in Fig. 9. By using the LWB smoothing function, the proposed model prevented zero probability. Hence, the temporal pattern was assessed by the proposed HLWBMM with a Brier score and calibration error of 0.1372 and 0.1893, respectively. Yet, the prevailing baseline models like HMM, MRF, SLDS, and DBN achieved a Brier score of 0.2572, 0.4849, 0.7183, and 0.8394 and calibration error of 0.3042, 0.5673, 0.7942, and 0.8894, respectively. Therefore, when compared to the traditional models, the proposed HLWBMM improved the temporal pattern estimation.

**Fig. 10:**
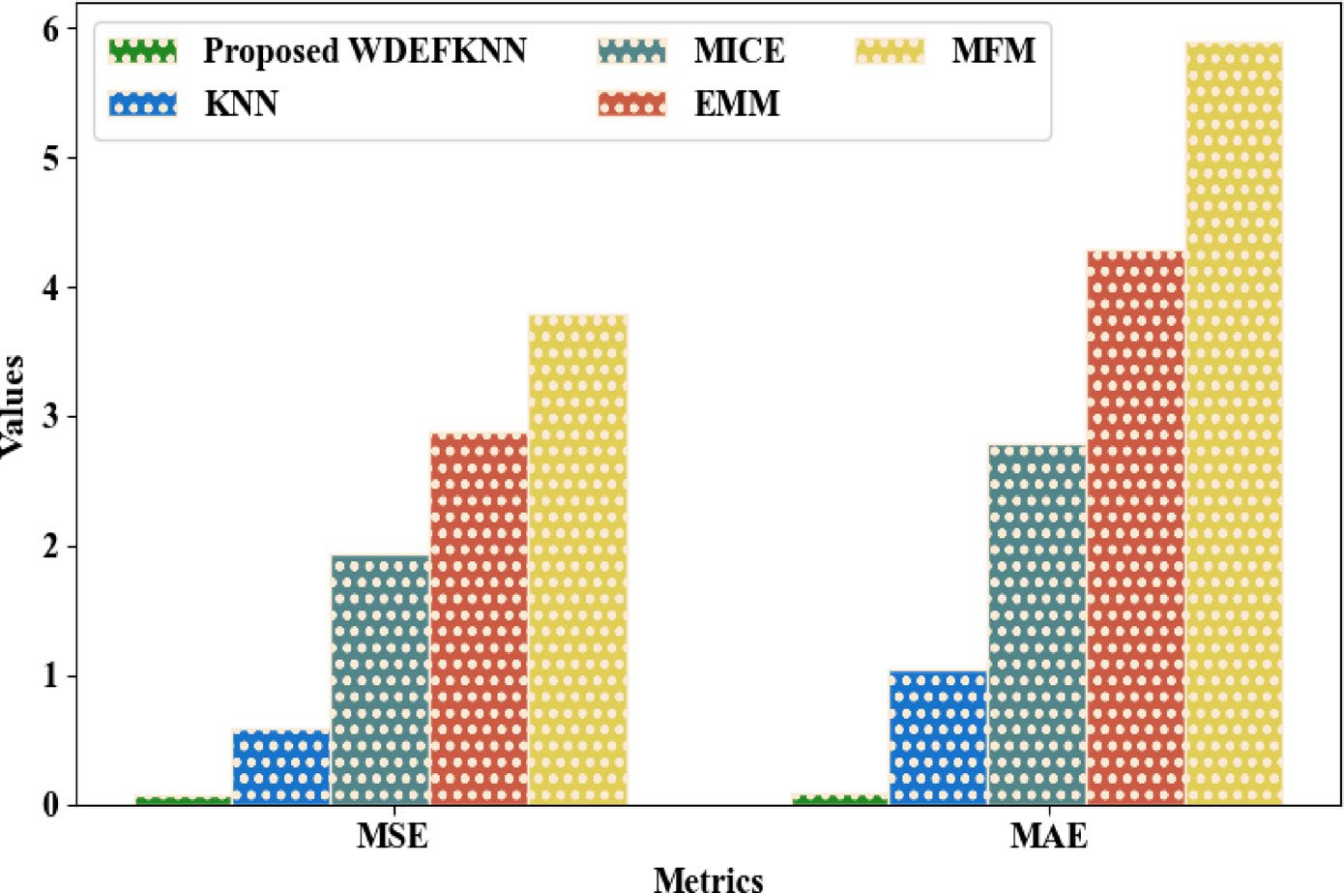
Graphical comparison of WDEFKNN.

The uncertainty owing to missing values was prevented by the proposed WDEFKNN with a Mean Squared Error (MSE) and a Mean Absolute Error (MAE) of 0.0682 and 0.0904, respectively. As depicted in Fig. 10, an MSE of 0.5932, 1.9421, 2.8831, and 3.7942, and an MAE of 1.0482, 2.7842, 4.2941, and 5.8931 were achieved by the traditional KNN, Multiple Imputation by Chained Equation (MICE), Expectation–Maximization Model (EMM), and Matrix Factorization Method (MFM), correspondingly. Thus, when contrasted with the existing approaches, the utilization of WDEF for determining the “K” factor enhanced the proposed model’s performance.

In Table 4, the proposed and the traditional methodologies’ comparison concerning intrusion detection is explained. Neural Network (NN), Random Forest (RF), SVM, Uncertainty Aware Dynamic Early Stopping Deep Belief Network (UA-DES-DBN), and CNN were used by the prevailing works for threat identification. The proposed work efficiently calculated the uncertainty and also examined the temporal patterns. Hence, an accuracy, precision, recall, and F-Measure of 99.299%, 99.301%, 99.256%, and 99.219%, respectively, were achieved by the proposed GRUSO-GRU. Nevertheless, the existing^31–33^ failed to overcome the uncertainty caused by missing values, thus achieving an accuracy of 98.6%, 83.795%, and 98.23%, and F-Measure of 97.1%, 81.859%, and 96%, correspondingly. Also, the traditional^34^ was unable to capture the temporal patterns, thus attaining an accuracy of 98.6%. Likewise, the prevailing^35^ caused low convergence, thus achieving a precision and a recall of 97% and 96%, correspondingly. Also, the existing^36^ used Convolutional Recurrent Neural Network (CRNN) for intrusion detection and attained a low accuracy, precision, recall, and F-Measure of 98.90%, 98.63%, 99.14%, and 99.03%, correspondingly. Additionally, the prevailing^37^ attained a very low accuracy of 97.05% by using the CNN Long Short Term Memory (CNNLSTM) technique. This depicted that the proposed methodology performed better in handling the uncertainties and identifying the intrusion in the network.

**Table 4:** Related works comparison.

Study	Methods	Accuracy (%)	Precision (%)	Recall (%)	F-Measure (%)
Proposed Work	GRUSO-GRU	99.299	99.301	99.256	99.219
^31^	NN	98.6	98.4	–	97.1
^32^	RF	83.795	82.203	83.795	81.859
^33^	SVM	98.23	94	98	96
^34^	UA-DES-DBN	98.6	–	–	–
^35^	CNN	96	97	96	96
^36^	CRNN	98.90	98.63	99.14	99.03
^37^	CNNLSTM	97.05	–	–	–

Table 5 presents the comparative analysis between the proposed classifier and the recent DL-based intrusion detection approaches evaluated on the CSE-CIC-IDS-2018 dataset. The proposed GRUSO-GRU obtained an accuracy of 99.299%, precision of 99.301%, recall of 99.256%, and an F-measure of 99.219% under a multi-class classification setting. In comparison, the TLDQN developed by^38^ reported an accuracy of 89%. Further, the existing Transformer model and the CNN-BiLSTM-Attention approach^39,40^ reported a precision of 86.3% and 95.17%, a recall of 86.3% and 94.26%, and an F-Measure of 85.3% and 96.28%, correspondingly. Overall, the proposed GRUSO-GRU demonstrated higher performance within the evaluated experimental configuration and by utilizing the CSE-CIC-IDS2018 dataset for NIDS.

**Table 5:** Comparison of existing works related to CSE-CIC-IDS2018 dataset.

Study	Models	Accuracy (%)	Precision (%)	Recall (%)	F-Measure (%)
Proposed System	GRUSO-GRU	99.299	99.301	99.256	99.219
^38^	TL-based Deep Q-Network (TLDQN)	89	–	–	–
^39^	Transformer model	–	86.3	86.3	85.3
^40^	CNN-BiLSTM-Attention approach	93.26	95.17	94.26	96.28

### Cross-dataset generalization analysis

In this section, cross-dataset generalization is done for the dataset used in the proposed work, namely CSE-CIC-IDS2018, and some other datasets related to intrusion detection, such as the Network Intrusion Detection Dataset and CIC-IDS-2017.

Table 6 depicts the cross-dataset generalization analysis of the proposed model and existing techniques across different NIDS datasets, such as CSE-CIC-IDS2018, Network Intrusion Detection Dataset, and CIC-IDS-2017. Here, the proposed GRUSO-GRU achieved a high accuracy, recall, and F-measure of 99.299%, 99.256%, and 99.219% for the CSE-CIC-IDS2018 dataset, 99.123%, 99.158%, and 99.021% for the Network Intrusion Detection Dataset, and 99.087%, 99.134%, and 99.011% for the CIC-IDS-2017 dataset. Here, improved performance is obtained owing to the usage of USO for elevating the learning efficiency and GR activation for avoiding the slow convergence. However, the prevailing GRU, Bi-LSTM, LSTM, and RNN attained a low average accuracy, recall, and F-Measure of 93.024%, 92.707%, and 92.158% for the CSE-CIC-IDS2018 dataset, 92.758%, 92.735%, and 92.534% for the Network Intrusion Detection Dataset, and 92.502%, 92.469%, and 92.295% for the CIC-IDS-2017 dataset. Thus, this cross-dataset analysis demonstrated that the proposed model was better than the prevailing techniques.

**Table 6:** Cross-dataset generalization validation.

Techniques	Accuracy (%)	Recall (%)	F-Measure (%)
*CSE-CIC-IDS2018*			
Proposed GRUSO-GRU	99.299	99.256	99.219
GRU	96.045	95.784	95.238
Bi-LSTM	94.146	93.984	93.352
LSTM	92.163	91.847	91.258
RNN	89.743	89.215	88.787
*Network intrusion detection dataset*			
Proposed GRUSO-GRU	99.123	99.158	99.021
GRU	95.987	96.012	95.872
Bi-LSTM	93.912	93.765	93.543
LSTM	91.702	91.657	91.402
RNN	89.432	89.509	89.321
*CIC-IDS-2017*			
Proposed GRUSO-GRU	99.087	99.134	99.011
GRU	95.762	95.812	95.634
Bi-LSTM	93.542	93.498	93.321
LSTM	91.409	91.376	91.216
RNN	89.295	89.192	89.012

## Conclusion

In this research, the uncertainty was computed, and the intrusion in the network was identified efficiently with multi-class classification and explainability. Firstly, the nodes in the network were initialized. Then, the data was sensed, and the NIDS was trained. To train the NIDS, the data was collected and pre-processed. Utilizing WDEFKNN, the missing value uncertainty was imputed at the time of pre-processing, thereby attaining an MSE of 0.0682. Further, by utilizing DBBDSSCAN, the behavioural pattern was analyzed, which attained a 0.928 Silhouette score. Then, the features were extracted. Afterward, the epistemic uncertainty was analyzed utilizing AA-NLS, which attained a 95.112 range consistency. In the meantime, utilizing HLWBMM, the temporal pattern was assessed with a 0.1372 Brier score. Further, GRUSO-GRU was used to identify the intrusion. The experiment evaluation on the CSE-CIC-IDS2018 dataset with full seven-class multiclass classification demonstrated 99.299% accuracy. Lastly, by utilizing LCFFOA, the alerts were prioritized for the attacked class with a 2289 ms latency for 100 nodes. All the reported performance values corresponded to multi-class classification under a stratified training and testing split, ensuring a realistic evaluation protocol. So, the proposed system shows effectiveness in detecting intrusion, thus improving cybersecurity in data transmission.

### Limitation of the study

The proposed system focused on managing the uncertainty caused by packet disorder. Yet, it did not ensure the complete security of users’ sensitive data during intelligence-sharing in network conditions. In a real-world network environment, data exchanged between nodes contained confidential information, and without strong encryption, authentication, and acess-control mechanism, the transmitted data could be tampered. Thus, the end-to-end confidentiality was reduced.

### Future scope

Thus, to overcome the data tampering during transmission, the future work will safeguard the security of the user’s sensitive data at the time of intelligence-sharing in network conditions. This further aids in improving the uncertainty computing.

## Data Availability

The datasets used in this study are publicly available intrusion detection datasets. The primary dataset used is the CSE-CIC-IDS2018 dataset, and additional validation was conducted using the Network Intrusion Detection Dataset and CIC-IDS-2017 dataset. These datasets are available through public repositories such as Kaggle. CSE-CIC-IDS2018 dataset: https://www.kaggle.com/datasets/solarmainframe/ids-intrusion-csv Network Intrusion Detection Dataset : [https://www.kaggle.com/datasets/chethuhn/network-intrusion-dataset] CIC-IDS-2017 dataset: [https://www.kaggle.com/datasets/sampadab17/network-intrusion-detection?resource=download].
